# Day-to-day regularity and diurnal switching of physical activity reduce depression-related behaviors: a time-series analysis of wearable device data

**DOI:** 10.1186/s12889-023-14984-6

**Published:** 2023-01-06

**Authors:** Satoshi Yokoyama, Fumi Kagawa, Masahiro Takamura, Koki Takagaki, Kohei Kambara, Yuki Mitsuyama, Ayaka Shimizu, Go Okada, Yasumasa Okamoto

**Affiliations:** 1 Department of Psychiatry and Neurosciences, Hiroshima University, 1-2-3 Kasumi, Minami-Ku, Hiroshima, 734-8551 Japan; 2 Hiroshima Prefectural Mental Health Center, Hiroshima, Japan; 3 Department of Neurology, Shimane University, Shimane, Japan; 4 Brain, Mind and KANSEI Sciences Research Center, Hiroshima University, Hiroshima, Japan; 5 Health Service Center, Hiroshima University, Hiroshima, Japan; 6 Faculty of Psychology, Doshisha University, Kyoto, Japan

**Keywords:** Time-series analysis, Physical activity, Wearable device, Depressive behavior

## Abstract

**Background:**

Wearable devices have been widely used in research to understand the relationship between habitual physical activity and mental health in the real world. However, little attention has been paid to the temporal variability in continuous physical activity patterns measured by these devices. Therefore, we analyzed time-series patterns of physical activity intensity measured by a wearable device and investigated the relationship between its model parameters and depression-related behaviors.

**Methods:**

Sixty-six individuals used the wearable device for one week and then answered a questionnaire on depression-related behaviors. A seasonal autoregressive integral moving average (SARIMA) model was fitted to the individual-level device data and the best individual model parameters were estimated via a grid search.

**Results:**

Out of 64 hyper-parameter combinations, 21 models were selected as optimal, and the models with a larger number of affiliations were found to have no seasonal autoregressive parameter. Conversely, about half of the optimal models indicated that physical activity on any given day fluctuated due to the previous day’s activity. In addition, both irregular rhythms in day-to-day activity and low-level of diurnal variability could lead to avoidant behavior patterns.

**Conclusion:**

Automatic and objective physical activity data from wearable devices showed that diurnal switching of physical activity, as well as day-to-day regularity rhythms, reduced depression-related behaviors. These time-series parameters may be useful for detecting behavioral issues that lie outside individuals’ subjective awareness.

**Supplementary Information:**

The online version contains supplementary material available at 10.1186/s12889-023-14984-6.

## Background

Healthy behavior can be understood as a sequence of habitual behaviors, and habit-related physical activities are predictive of mental health [1]. For example, an engagement in moderate-to-vigorous physical activity (MVPA), such as brisk walking, during the past seven days, reduces the odds of experiencing depression among adolescents [2]. Additionally, it is known that overall daily physical activity patterns, low MVPA, and high sedentary behavior influence depression [3]. These studies have used total or mean values for intensity or duration of a physical activity. Furthermore, these were subjective and often focused on an activity domain of interests, including leisure time, household, transportation, and work [2, 4, 5]. Therefore, objective and comprehensive information about habitual physical activity in daily life has not been accurate enough with respect to investigating mental health issues, owing to overly compressed and subjective information prone to memory ​bias [6]. The development of technologies for highly accurate measurements without subjective biases will enhance our understanding of the association between habitual behavior and mental health.

Here, understanding about relationship between physical activity and depression is often used in behavioral activation in a clinical application, which is a psychotherapy to habituate specific behaviors intended to increase contact with positive reinforcements [7]. Depressive individuals encounter less positive reinforcement and frequently engage in depression-related behaviors such as avoidance and rumination to avoid the distress [8]. These maintenance and exacerbation mechanisms are usually explained by functional analytical models that focus on a certain unit of behavior. Therefore, there is no precise correspondence between empirical data and its theory about when, for how long, and how to perform the behaviors in daily behavioral transitions. If we can realistically measure the relationship between daily behavioral patterns and depression-related behaviors, we may be able to uncover health-promoting mechanisms in behavioral theory of depression.

Certain technological advances have enabled us to measure the sequential intensity of physical activity using wearable devices such as a smart watch. These allowed researchers to conclude an association between physical activity and health risk without the problem of subjective reporting [9, 10]. A current concern is the lack of an analytic strategy to extract relevant information from the large amount of time-series raw data generated by the device, compared to the improvements in data collection technology [11]. One solution is to use machine learning-based pattern recognition to classify physical activity, which is useful in detecting mental health problems by automatically labeling to physical activity [12]. However, the recognized patterns are often remained in the black box. While this does not interfere with the motivation for prediction-oriented machine learning models, it does make it difficult to understand the rationale behind the predictions [13]. Therefore, the relationship between habitual physical activity patterns in daily life and depression-related behaviors remains unclear. A better understanding of this mechanism will help us detect the behavioral patterns related to depression, and allow us to develop clinical ideas for improving their discomfort. Digital wearable devices generate a vast amount of high-frequency high-dimensional time-series data that require new methods of analysis [14].

Among the many time-series models being proposed, the Autoregressive Integrated Moving Average (ARIMA) model is the most widely used and well-known [15]. ARIMA is a linear model for time-series forecasting that expresses the difference in time-series data using autoregressive (AR) and moving averages (MA) parameters [16]. The seasonal ARIMA (SARIMA) model [16], which adds cyclicality to the ARIMA model, may better reflect the habituation of the physical activities that people perform in a day-to-day cycle (24 h). In particular, the two seasonal parameters in the 24-h cycle model, seasonal AR (sAR) and seasonal MA (sMA), indicate habituation in terms of the effect of the previous day's physical activity on current physical activity. However, to the authors' knowledge, no report has examined the physical activity habituation in wearable device data using the SARIMA model.

The current study aimed to extract habitual physical activity patterns from sequential data obtained from wearable devices using the SARIMA model with a 24-h cycle, and to examine the relationship between these patterns and depression-related behaviors, as an indicator of mental health, in the real world.

## Methods

### Participants

Sixty-six volunteers (mean age = 21.7 ± 1.60; females = 24; BMI = 20.84 ± 2.57) were recruited from among the students of Hiroshima University. All participants were attending school without disability and further that they were not currently receiving psychiatric treatment. They attended a research briefing and provided written informed consent for participation. Accelerometers were distributed on the first day of the study, and physical activity was measured continuously for 165 h from midnight the next day to 21:00 on the eight day. After the completion of the measurement, the experimenter recovered the device and extracted the recorded data. Participants’ personal information was protected because the device recorded only 3-axis acceleration and did not have any information about their lives, such as location (where they stayed, or how far they traveled). The collected participants (n = 66) were designed as follows: an estimated number of 52 cases required for the multiple regression analysis using effect size (f^2^ = 0.35), significant level (alpha = 0.05), and power (beta = 0.8), with an assumed measurement miss rate of 20%.

### Physical activity

Participants were asked to wear the Device Arm Triaxial Accelerometry system (UW-301BT Life Log, Hitachi Ltd, Tokyo) on their non-dominant wrist for seven days, except while bathing. This device can record 3-axis acceleration with a 20 Hz sampling rate [17]. The recoding signal was integrated to calculate the intensity of physical activity at each minute, referred to as metabolic equivalents (METs), via conversion algorithms [18, 19] provided in the device. The device has been confirmed to detect physical motions that correlate with actual frequency of physical movement [20], and used for in-home measurement of rehabilitation effectiveness [21] and extraction of episodes corresponding to behaviors in daily life [22].

As one MET is equivalent to the energy cost of sitting quietly, we were able to assume zero MET as non-wearing time such as bathing. We defined a missing period as any non-wearing time of more than 30 consecutive minutes. Since 22 participants had the defined missing period, 44 (mean age = 21.5 ± 1.02; females = 18; BMI = 20.9 ± 2.94) were included in the analysis excluding them (statistical power for a primary regression analysis = 0.81). And data for non-wearing time of less than 30 min were substituted with the mean of the previous 15 min. The complemented data were totaled every hour, leading to a total of 165 time points (since the mounting start time was uneven on the first day, midnight of the second day was the measurement start time). This multiplication of the MET value by the engaged time is a continuous variable of the physical activity level called "MET-minutes" and corresponds to the consumed calories (Kcal) for a person weighing 60 kg [23]. The total intensity of hourly physical activity was entered into the SARIMA model. In clinical practice, therapists often ask patients with depression to record hourly activities in order to understand their behavioral patterns [24, 25]. As behavioral activation interventions also propose an "hour-by-hour basis" account for activity [26], examining variability in this time unit, hour-by-hour, activity could have been useful for clinical application of this study.

## Questionnaires

### Japanese version of Beck's Depression Inventory-II (BDI-II)

The BDI-II is a widely used scale for assessing depressive symptoms, and consists of 21 self-report items. The items are scored using a 4-point scale. The higher the score, ranging from 0–63 points, the more severe the disease, with 14 points or more generally being the cutoff for having symptoms [27]. The Japanese version of the scale has demonstrated reliability (Cronbach’s alpha = 0.87) and validity (criterion related validity with other depression scale: r = 0.69) [28].

### Japanese version of the Behavioral Activation for Depression Scale (BADS)

The BADS [29] measures depression-related behavior patterns based on behavioral theories of depression. To facilitate the translation of the findings obtained in this study into an understanding of the behavioral theory of depression and intervention techniques such as a behavioral activation, we used this scale, which can capture the behavioral characteristics of people with depression. It consists of 25 items rated on a 7-point scale (0: Not at all to 6: Completely). The four subscales of the BADS are Activation (AC: 7 items) representing goal-directed activation and completion of scheduled activities; Avoidance/Rumination (AR: 8 items) representing avoidance of negative aversive states and engaging in rumination; Work/School Impairment (WS: 5 items); and Social Impairment (SI: 5 items). The Japanese version of the BADS has demonstrated reliability (Cronbach’s alpha = 0.78) and validity (criterion related validity with depression scales: r = -0.68 to -0.71) [30].

## SARIMA model

A SARIMA model is formed by including seasonal elements in the ARIMA models [31]. The terms for non-seasonal and seasonal elements in a SARIMA model are as follows: SARIMA(p, d, q, P, D, Q, m), where, p, d, and q are non-seasonal delays of AR type, non-seasonal delays of MA type, and non-seasonal integration order, respectively. AR (p) means that the past values of itself until t-p time points are included as predictor variables of value at t point, and MA (q) means that the noise values until t-q time points are also included as predictor variables of value at t point. Then, the dependent variable is usually integrated for stationary form by a sequence of differences from the value at t-d time. The SARIMA model includes the seasonal element as a hyper-parameter (P, D, Q, m), where, P, D, Q, and m are seasonal delays of AR type, seasonal delays of MA type, seasonal integration order, and length of seasonality cycle, respectively. Since people are usually active in 24-h cycles, we fixed the parameter m to 24 (24 h), that is SARIMA (p, d, q, P, D, Q, 24) model, to obtain time-series information about daily habitual physical activity.

### Grid search for an individual optimal model

We performed a grid search of the model order values, (p, d, q, P, D, Q, 24), for all parameter combinations for each individual. In order to narrow down the range of candidate parameters, we asked participants to report their average daily physical activity time (“On average, how much time in total did you usually spend doing physical activities on one day?”). This inquiry was guided by the internationally standardized physical activity questionnaire, Japanese International Physical Activity Questionnaire Environmental Module [32]. Although the total activity time perceived by the participants may not necessarily be continuous, it was used as information to indicate the approximate range of time spent in a certain activity. Since the mean value was 2.3 h (mode = 2.0 h), the range of influence of past physical activity was limited to 0–3 h. Hence, there were four candidate values for the hyper-parameters (p, q) between 0 and 3, respectively. The seasonal hyper-parameter determines the influence of the 24-h cycle. To specify whether the previous day's cycle should be included in the model, the candidate hyper-parameters (P, Q) were denoted by 0 or 1. The hyper-parameters (d, D) that specify the range of the difference were set to 1 in order to reduce the calculation cost. We tried to include the distinction between weekdays and holidays as an exogenous regressor in the model, but this had no effect and did not change the goodness of fit of the model. Therefore, we did not include a regressor for the weekend in the model, but estimated it using fewer explanatory variables.

Finally, the number of candidate combinations for hyper-parameters of SARIMA (p, d, q, P, D, Q, 24) was 64 (4 × 1x4 × 2x1 × 2x1). After fitting all candidate models to the data, a model with the lowest Akaike's information criterion (AIC) was determined as the optimal model a participant.

### Interpretability of model parameters

From the individuals' optimal model, we obtained the weight estimates of each term, AR(p), MA(q), sAR(P) and sMA(Q), as indicators that reflected the time-series information of physical activity. Our SARIMA models had fixed the seasonal/non-seasonal difference to one (i.e. d = D = 1). Therefore, each weight estimate of the parameters explained the change in physical activity intensity on a daily or hourly basis.

Two seasonal parameters, sAR and sMA, may reflect daily habituation. When *P* = 0, sAR has no weight, which reflected that “changes” in activity are not affected by the previous day's activity. If the hyper-parameter *P* = 1, the sAR(1) weights reflected that daily physical activity varied depending on the previous day's physical activity. Therefore, unlike *P* = 0, the physical activity pattern fluctuated (increased or decreased) from day to day, which indicated unstable habitual behavior (irregularity). The weights of sMA(1) reflected the degree to which the change in physical activity from the previous day could be explained by the residual of the predicted value due to the past physical activity. When this weight value was high, the increase in activity from the previous day was proportional to the change in the previous day, but it depended on the non-linear increase in the previous day. Therefore, the parameter sMA also indicated the unstable physical activity patterns from day to day. The non-seasonal parameters indicated whether activity in the closer past (here, 0 to 3 h ago) influenced the change in one-hour interval.

### Statistical analysis

We performed regression analyses for the scores of each questionnaire using the weights of the parameters in the SARIMA model as explanatory variables. In order to maintain analytical power, we avoided grouping or selection based on optimal models that would identify participants who did not have the order of the parameter (e.g., *p* = 0). Instead, we substituted the weight of the non-existent parameter as zero in the regression model. In this analytical model, multicollinearity was assessed using variance inflation factor (VIF). The significance threshold was set at p < 0.05. All analyses were done via the Python library “statsmodels” (ver.0.11.0).

## Results

### Selection of optimal SARIMA models for individuals

Out of the 64 candidate models based on combinations of the orders of the seven hyper-parameters, 21 models were estimated as optimal models with the lowest within-individual AIC in at least one participant. The physical activity records of the members belonging to each model are illustrated in Supplementary figure (Figure S1). The model numbers are a formality and do not reflect superiority or inferiority.

**Fig. 1:**
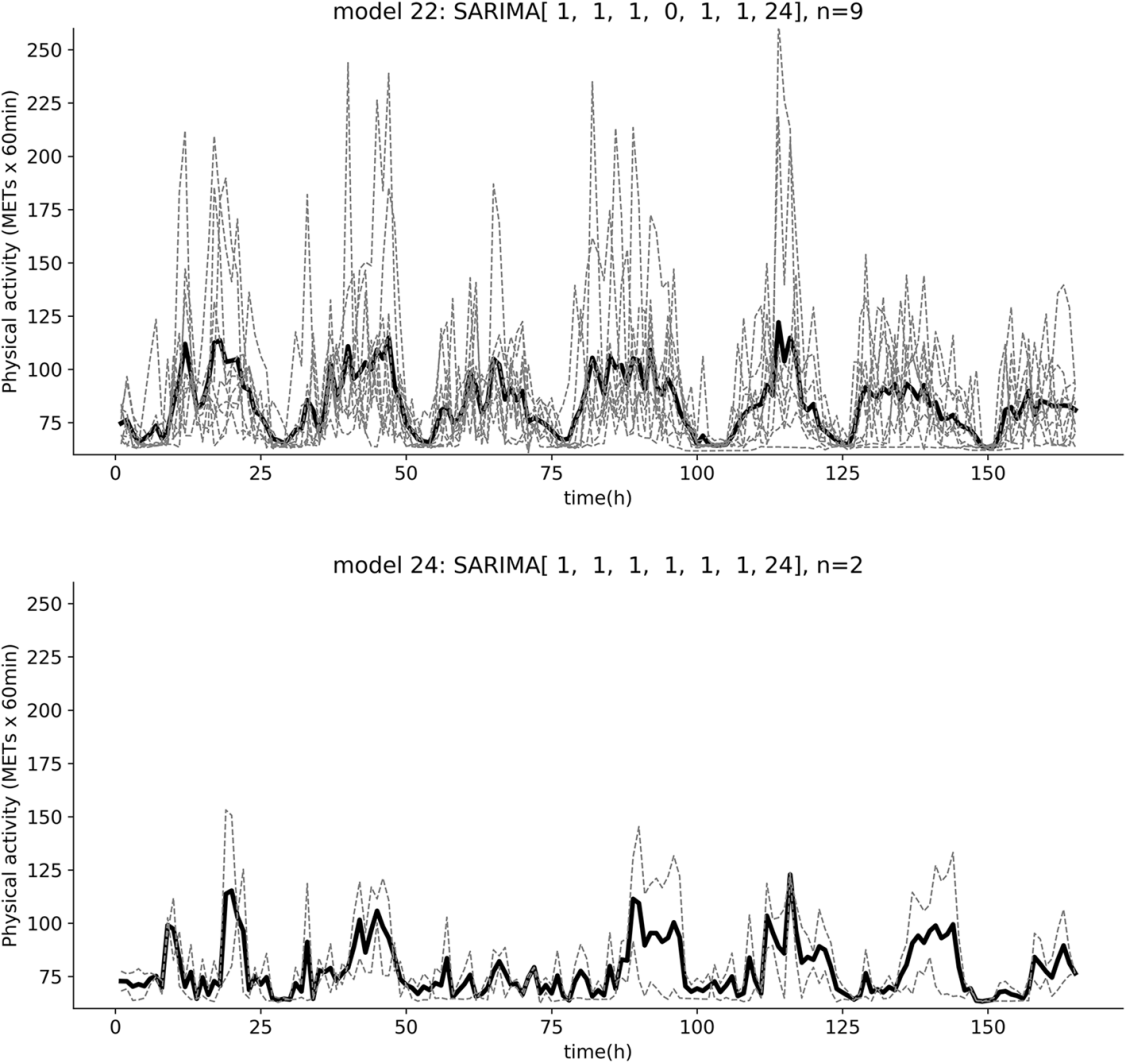
Time-series plot of the physical activity of participants belonging to representative models. Note: The dashed lines show the physical activity of each participant, and the solid lines show their mean values. The model with the most affiliations (model # 22) is shown in the upper panel. The lower panel is a comparison model that differs only in P from the upper panel (model # 24). All optimal models and their physical activity are shown in Supplementary Figure (Figure S1)

The results for nine participants (20.5%) showed SARIMA (1, 1, 1, 0, 1, 1, 24), model #22, as the best model, and this model was selected most frequently (Fig. 1: top panel). SARIMA (3, 1, 3, 0, 1, 1, 24), model #62, and SARIMA (2, 1, 2, 0, 1, 1, 24), model #42, were selected by five (11.4%) and four (9.1%) participants, respectively (Supplementary Figure: Figure S1). These high-ranked models took zero as the seasonal autoregressive parameter, sAR (*P* = 0), and orders for the non-seasonal parameters, AR and MA, in the model were of equal value (*p* = q). Comparing the time-series plots between models with the same hyper-parameters other than P (e.g., model #6 vs model #8; model #22 vs model #24), we can visually assess that the model with *P* = 0 was more stable in the diurnal cycle (Fig. 1 and Figure S1). High-ranked models had *P* = 0, indicating that a majority of college students (65.9%) were engaged in habitual physical activity.

Whereas, the number of models with *P* = 1 was 11 (model #7, 8, 12, 15, 16, 23, 24, 44, 56, 63, 64) out of the 21 optimal models obtained, and these were judged to be the optimal models for 15 participants (34.1%).

### Relationships between the weights of parameters and depression-related behaviors

**Fig. 2:**
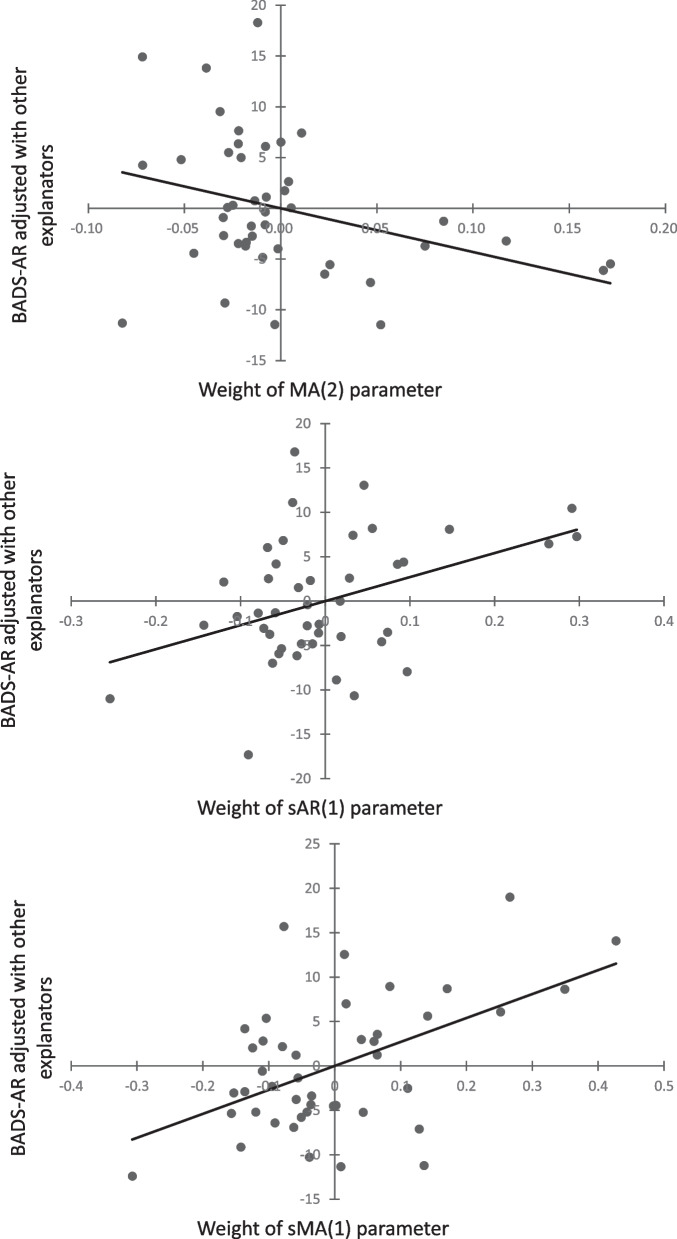
Partial residual plots between significant explanatory variables and the BADS-AR. Note: The values on the axes show the centered partial residuals. sAR = seasonal auto-regression, MA/sMA = non-seasonal/seasonal moving-average, BADS-AR = Avoidance/Rumination subscale of the Behavioral Activation for Depression Scale (BADS)

Only the regression model for the BADS-AR was supported by the significance test (*R*^*2*^ = 0.34, *F*(8,35) = 2.28, *p* < 0.05). The estimation results for all the regression models and the partial residual plots for significant explanatory variables on the BADS-AR are shown in Table 1 and Fig. 2.

**Table 1:** Results of regression analyses for the weights of model parameters on depressive symptoms and depression-related behaviors

	Dependent variables				
	BDI-II	BADS-AC	BADS-AR	BADS-WS	BADS-SI
AR(1)	0.27	-0.70	0.73	1.25	0.20
AR(2)	-0.16	0.21	-0.13	0.11	-0.47
AR(3)	-0.30	0.03	-0.11	-0.01	-0.10
MA(1)	-0.27	1.64	-2.99	-0.26	-0.25
MA(2)	-0.41	1.69	-2.71 ^*^	-0.91	-0.43
MA(3)	-0.22	1.38	-2.01	-0.85	0.07
sAR(1)	0.25	-0.18	0.75 ^*^	1.03	-0.09
sMA(1)	0.38	-0.34	1.03 ^*^	0.98	0.16
R^2^	0.11	0.14	0.34	0.33	0.12
F	0.53	0.69	2.28 ^*^	2.15	0.60

Note: *AR* Auto-regression parameter, *MA* Moving-average parameter, prefix, *“s”* seasonal, *BDI-II* Beck Depression Inventory-II, *BADS* Behavioral Activation for Depression Scale, *BADS-AC* BADS Activation subscale, *BADS-AR* BADS Avoidance/Rumination subscale, *BADS-WS* BADS Work/School Impairment subscale, *BADS-SI* BADS Social Impairment subscale. The VIF values for all explanatory variables were less than 3.29, which indicated that multicollinearity concerns were too small

^*^p < 0.05

Significant positive regression coefficients for the weights of sAR(1) (coefficient = 0.75, *p* < 0.05) and sMA(1) (coefficient = 1.03, *p* < 0.05) indicate that irregular day-to-day activity patterns related to more avoidance and rumination behaviors. Conversely, the regression coefficients for MA(2) (coefficient = -2.71, *p* < 0.05) weight was a significant negative value, suggesting that sudden changes in total physical activity in the 2-h-old epoch related to reduction of depression-related behaviors such as avoidance and rumination.

## Discussion

In this study, we extracted habituation data by modeling participants’ real-life physical activity time course. The results of model selection showed that more than 60% of college students had stable physical activity patterns that were not affected by the previous day. However, not all college students necessarily had a regular daily routine. Based on the results of the regression analysis, irregular day-to-day activity patterns may be associated with an increase in depression-related behaviors such as avoidance and rumination. Interestingly, we observed that the obvious modification/switching of physical activity patterns within the epoch length of 2 h prior was likely to be linked to healthy physical activity. Consistent with the theory of psychological interventions [8, 33], unmodified activity patterns during the day and lack of moderate rest may be associated with increased depression-related behaviors. While much evidence has already shown a relationship between lifestyle rhythms and depression based on limited measurements or cognitive experiments [34–36], this is the first study that has demonstrated it using temporal characteristics of real-world physical activity.

Irregular social rhythm has been individually noted as a negative predictor of mental health in several life domains including meals, social contacts, and sleep and wake times [37]. These well-known findings have focused on a subset of lifestyles and do not represent the overall regularity of their lives. Previous studies using accelerometers have the similar concerns. They had cut out the duration or frequency of a specific intensity range (e.g., ≥ 3.0 METs) after continuously measuring physical activity with accelerometers [38, 39]. However, some life domains are often continuous and complementary. For example, a man who is out on his usual day off enjoys a meal in a restaurant on a rainy day is not consistent in content (i.e., irregular activity), but his “going out” is complemented by another behavior. This could be a quantitatively regular activity pattern, although the content and frequency/duration of individual activity were different. Therefore, measuring the frequency and duration of individual physical activities is not equivalent to knowing the rhythmic variability of overall physical activity. Our study revealed a simplified weekly variation in the intensity of physical activity without subjective ratings, instead of breaking it down into separate behavioral domains. Regardless of what behaviors the participants engaged in or what behavioral rhythms they disrupted, less day-to-day variability contributed to a reduction in depression-related behaviors such as avoidance and rumination. Our findings provide a perspective of overall lifestyle adjustment, supporting many of the previous findings on lifestyle rhythms and mental health.

It is important to highlight the implications of depressive behaviors in this sample of this study. Avoidance and rumination behaviors frequently predict depression even in non-clinical samples such as the participants in this study, and their effects are sometimes more powerful than that of anxiety, which is well known to be associated with depression [40]. Avoidance behavior seeks to decrease distress in life situations where a person perceives negative consequences, and rumination refers to repetitive and continuous negative thinking [8]. In college life, where there is little coercion, it may be assumed that experiencing failure, in academic or interpersonal relationships, for example, will lead to a change in behavior patterns, such as absences from school or part-time jobs, to avoid the negative situation. In fact, irregular daily life is one of the sub-scales on a mental health scale among Japanese university students [41]. In addition, repetitive thinking about a negative experience may inhibit positive/active behaviors, consequently, changes behavioral patterns. In this manner, these depression-related behaviors may emerge as inconsistent activity patterns, and this irregularity predicts students’ mental health problems.

In a shorter time span such as diurnal variability, the result revealed that pronounced modified physical activity patterns during the previous two hours could be associated with fewer depression-related behaviors. In our SARIMA model, the MA is a discontinuous, sudden, change in physical activity that cannot be predicted by past physical activity. Considering that physical activity compressed into a 1-h epoch length in this study, this parameter may reflect a pronounced switching in overall patterns rather than minute variations in activity. Many young people have been reported to spend more than two hours per day in screen viewing and sedentary activities [42–44]. These hours may include minor activity variations such as going to the toilet or eating and drinking, but rest activities are aggregated into larger time lengths. The integrated activity changes within the second time epoch represented by MA(2) may reflect the beginning or closing of these overall rest periods. In any case, this finding regarding the sudden switching of activities within this time window provides clinical implications for focusing on the two-hourly switching of activities on the habitual activity rhythm. Our results did not show that these large-scale changes in activity patterns at pats 1 or 3 h were associated with avoidance or rumination. Therefore, drastic changes in activity at every hour or after a longer period than 2 h would not be very meaningful on depressive behaviors. In the future study, elaboration of a person's continuous activity variations will further clarify the meaning of the second hour.

## Limitations

The present study has several limitations. First, we summarized physical activity in terms of total METs per hour, whereas several previous studies have reported that minute-long activity promotes health [38, 39]. Since we prioritized clinical applications, assessing activity on an "hour-by-hour basis" was close to the clinically method. However, it should be emphasized that various physical activities and minor variations were compressed into the one-hour period. Second, sampling only a non-clinical group may limit the interpretation of the association with the BADS-AR. Although the present study focused on depression-related behaviors in individuals with no current psychiatric disorders, future studies among patients with depression may be needed. The choice of population is very narrow and the fact that it is recruited from a normal population with limited mental issues is a challenge. Finally, participants used the wearable device for only a week. Further understanding of habitual behavior demands more long-term measurement. However, even with the one-week period, there was a large amount of missing data, as in other studies using wearable devices. Further research may be needed to understand physical activity patterns over a longer cycle, after issues such as device management and continuous data sampling are resolved.

## Conclusion

In summary, the objective data automatically measured by the wearable device showed that regular day to day rhythms and moderate diurnal switching of physical activities related to reduction of depression-related behaviors such as avoidance and rumination. In addition, this study clarified the relationship between time-series patterns of physical activity and depression-related behaviors, providing insights not only into the detection of mental health problems but also the measures to improve them. It may accelerate the delivery of care by detecting the likelihood of depression-related behaviors that are outside individuals’ subjective awareness.

## Supplementary Information

**Additional file 1: Figure S1.** Time-series plots across 165 hours for the physical activities of the participants belonging to each model.The model number and the number of participants belonging to the model are shown in the title of each plot. The dashed lines represent the physical activities of individuals, and the solid lines are their average values. If there is only one person in a model, the two lines appear to overlap. Note that the model numbers are a formality and do not reflect superiority or inferiority.

## Data Availability

The datasets generated and/or analyzed during the current study are not publicly available due to do not have consent from all patients to publish this data, but are available from the corresponding author on reasonable request.

## Abbreviations

ARIMA: Autoregressive Integrated Moving Average model
SARIMA: Seasonal Autoregressive Integrated Moving Average model
BDI-II: Beck's Depression Inventory-2^nd^ edition
BADS: Behavioral Activation for Depression Scale
