# Supplementary material for: Day-to-day regularity and diurnal switching of physical activity reduce depression-related behaviors: a time-series analysis of wearable device data
Source: BMC Public Health. 2023 Jan 6;23:34. doi: 10.1186/s12889-023-14984-6 (PMC9817381; doi:10.1186/s12889-023-14984-6)

**Supplementary information for “Day-to-Day regularity and diurnal switching of physical activity reduce depression-related behaviors: A time-series analysis of wearable device data.”**

Satoshi YOKOYAMA^1^, PhD., Fumi KAGAWA^2^, MD, Masahiro TAKAMURA^3,4^, PhD., Koki TAKAGAKI^5^, PhD., Kohei KAMBARA^6^, PhD., Yuki Mitsuyama^1^, MA, Ayaka Shimizu^1^, MD, Go OKADA^1^, MD, PhD., Yasumasa OKAMOTO^1^, MD, PhD.

1. Department of Psychiatry and Neurosciences, Hiroshima University, Japan

2. Hiroshima Prefectural Mental Health Center, Japan

3. Department of Neurology, Shimane University, Shimane, Japan

4. Brain, Mind and KANSEI Sciences Research Center, Hiroshima University, Hiroshima, Japan

5. Health Service Center, Hiroshima University, Japan

6. Faculty of Psychology, Doshisha University, Japan

**Corresponding author:** Yasumasa Okamoto M.D. Ph.D. (oy@hiroshima-u.ac.jp)

**Supplementary information**

**Supplementary figures.**

Figure S1

**Figure S1: Time-series plots across 165 hours for the physical activities of the participants belonging to each model.**

The model number and the number of participants belonging to the model are shown in the title of each plot. The dashed lines represent the physical activities of individuals, and the solid lines are their average values. If there is only one person in a model, the two lines appear to overlap. Note that the model numbers are a formality and do not reflect superiority or inferiority.


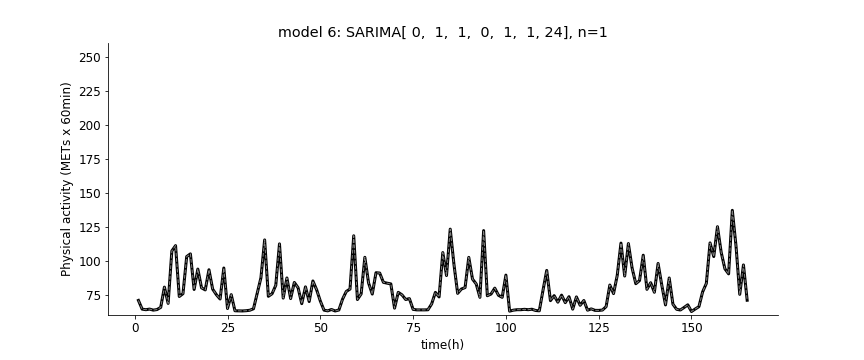

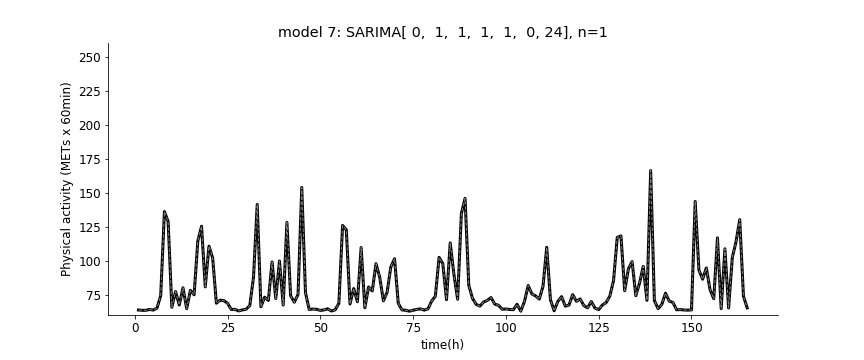

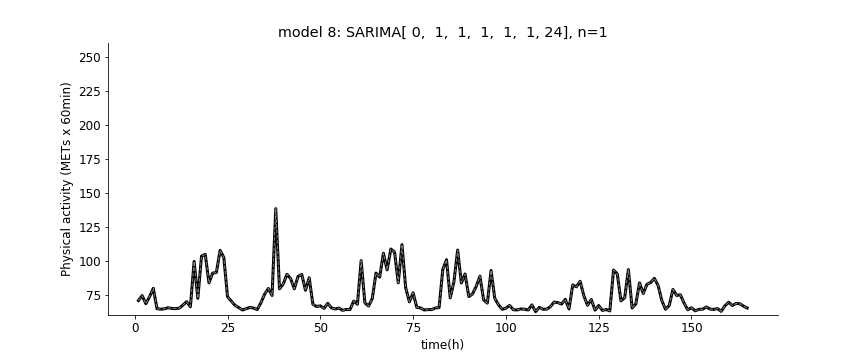


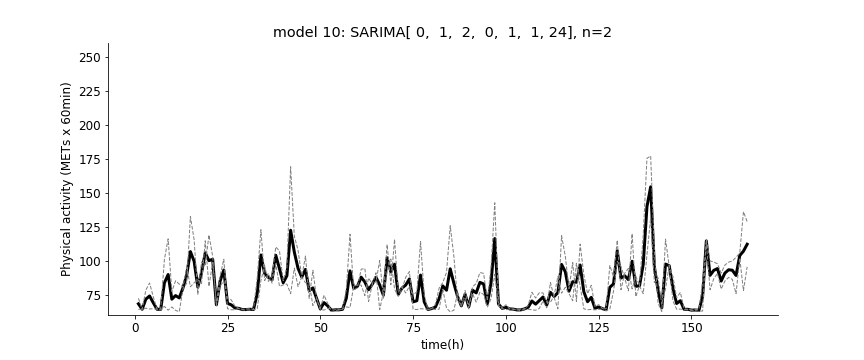


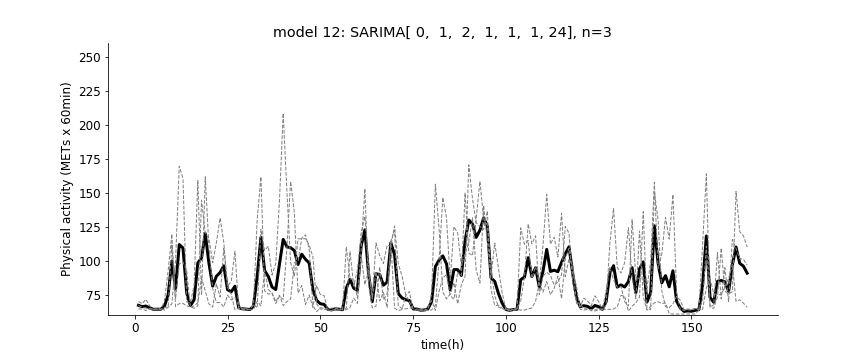

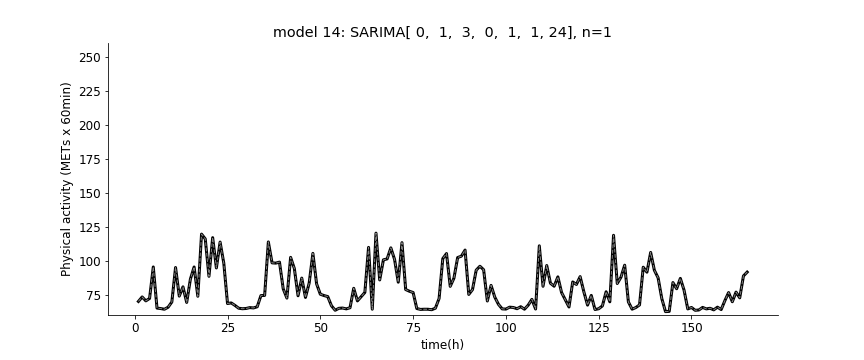

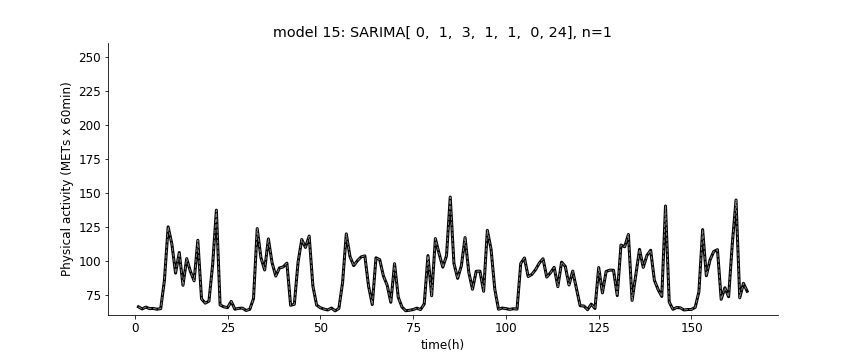

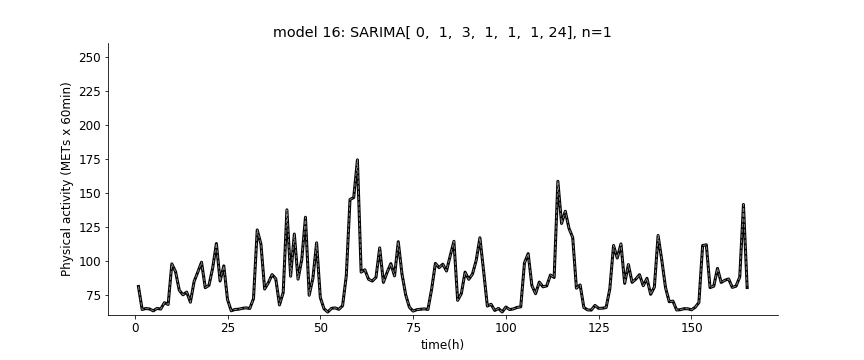

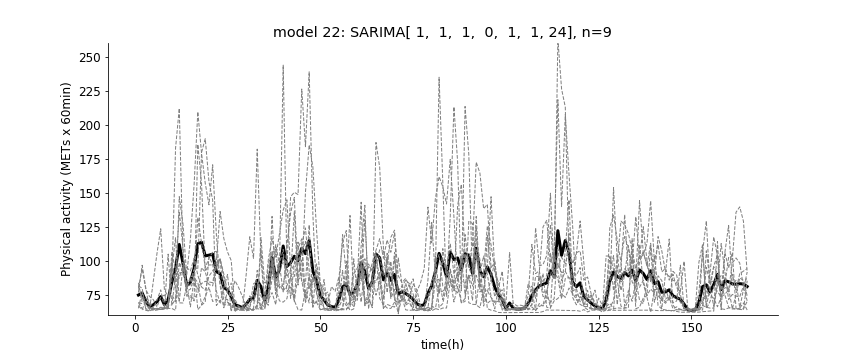

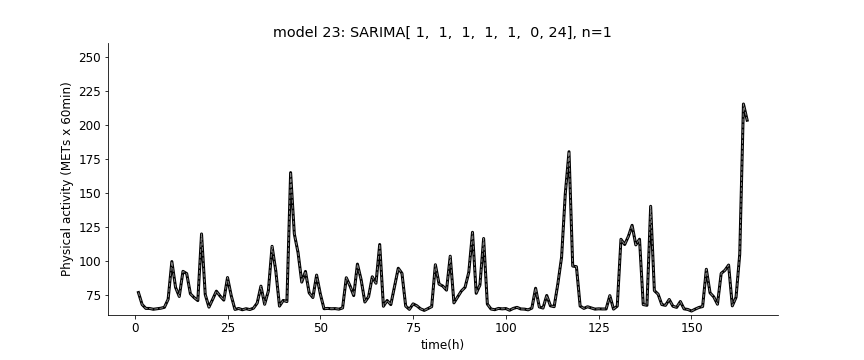

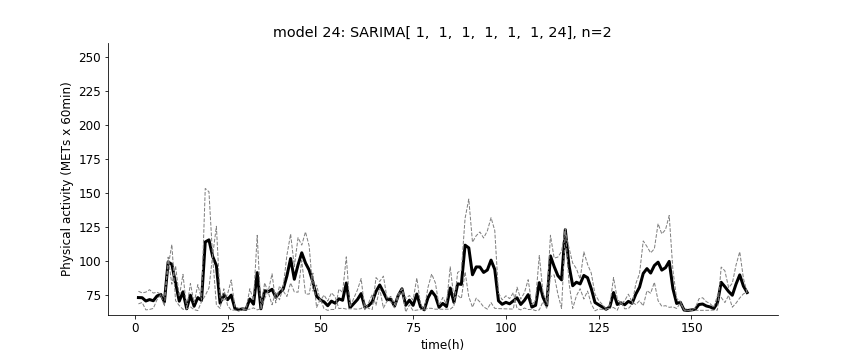

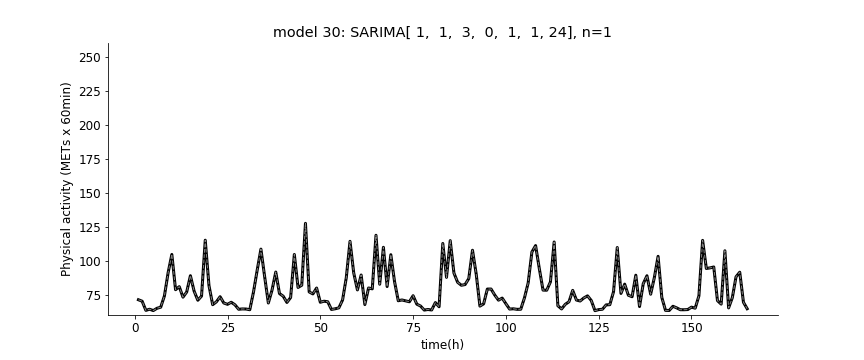

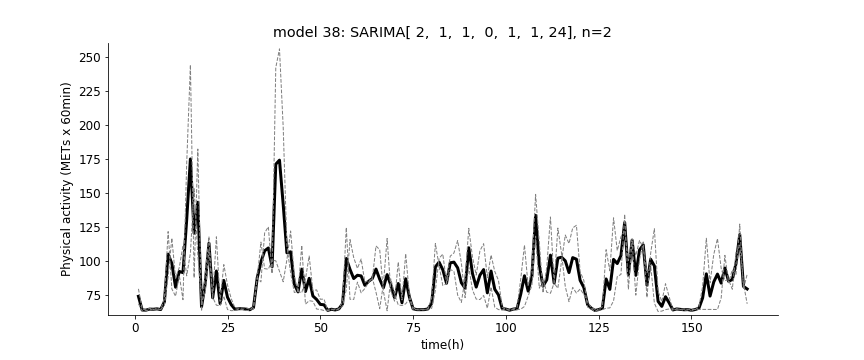

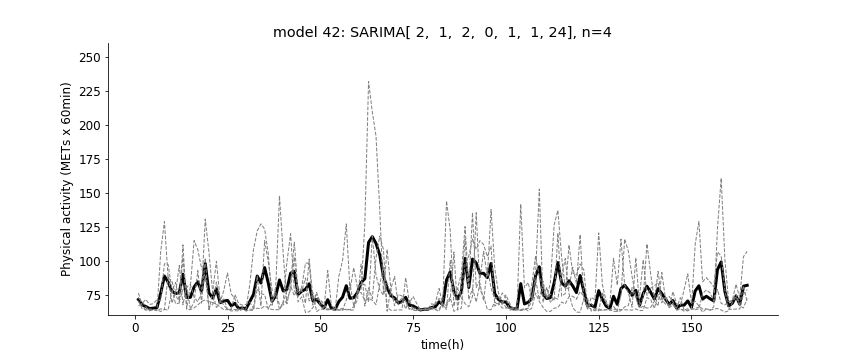

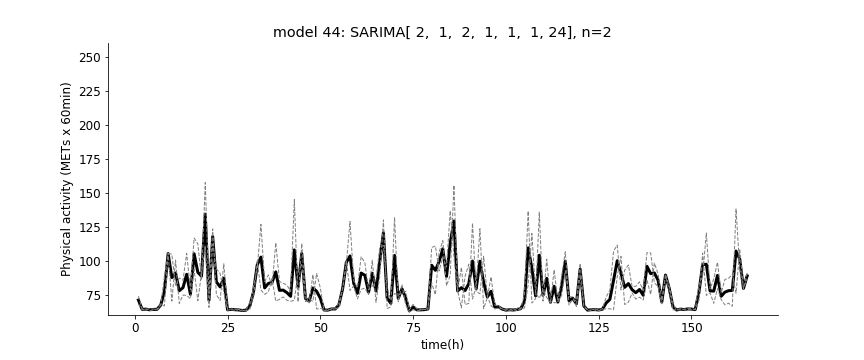

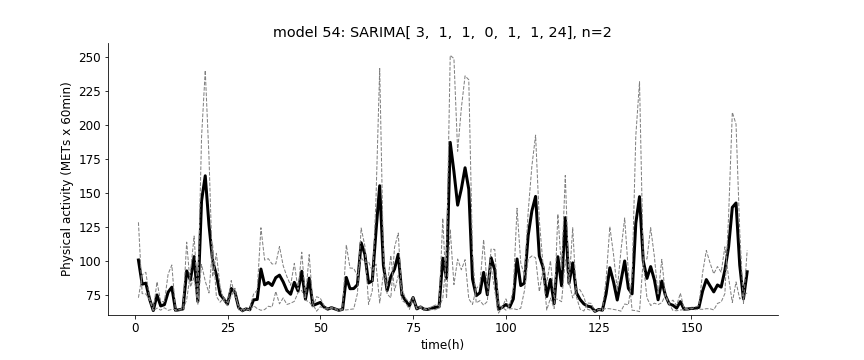

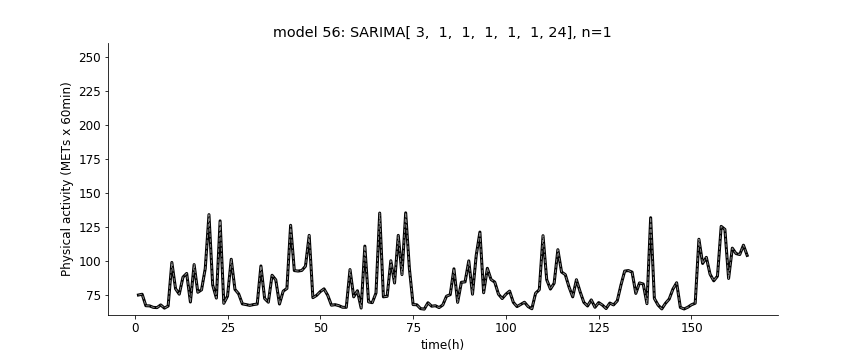

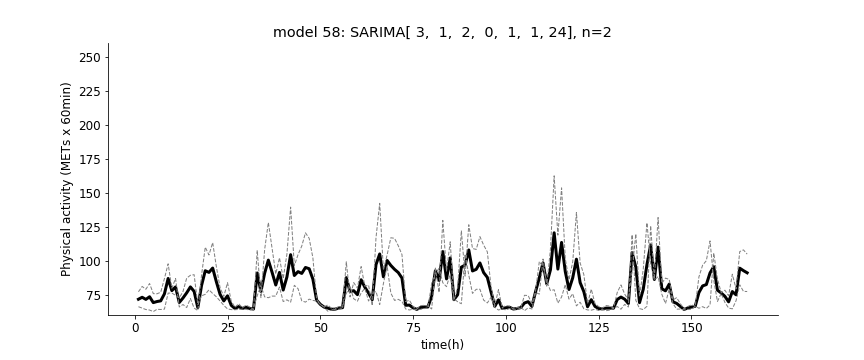

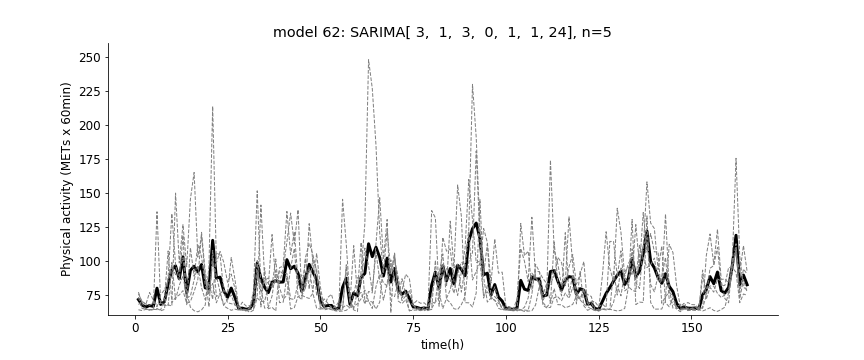

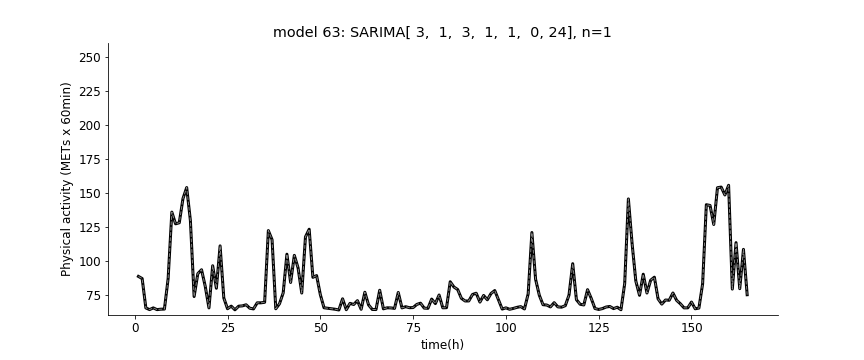

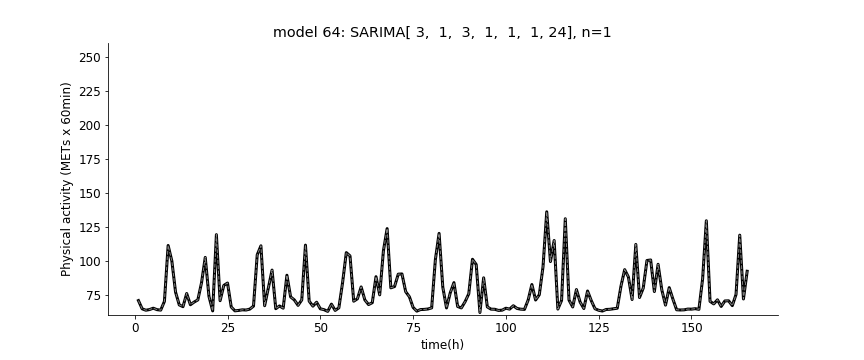

Supplement: Supplementary file 1 — Additional file 1: Figure S1. Time-series plots across 165 hours for the physical activities of the participants belonging to each model.The model number and the number of participants belonging to the model are shown in the title of each plot. The dashed lines represent the physical activities of individuals, and the solid lines are their average values. If there is only one person in a model, the two lines appear to overlap. Note that the model numbers are a formality and do not reflect superiority or inferiority. [file 12889_2023_14984_MOESM1_ESM.docx]
